# Effects of urbanisation and seasons on the relationship between frozen road conditions and road traffic injury: a longitudinal study of national emergency medical service data in South Korea

**DOI:** 10.1136/ip-2024-045327

**Published:** 2024-12-04

**Authors:** Jun Hee Won, Jaehong Yoon, Joo Jeong, Younshik Chung, Sangjin Han, Young Sun Ro, Ja-Ho Leigh

**Affiliations:** 1 Department of Rehabilitation Medicine, Seoul National University Hospital, Seoul, Korea (the Republic of); 2 National Traffic Injury Rehabilitation Research Institute, National Traffic Injury Rehabilitation Hospital, Yangpyeong, Korea (the Republic of); 3 Department of Emergency Medicine, Seoul National University Bundang Hospital, Seongnam, Korea (the Republic of); 4 Laboratory of Emergency Medical Services, Seoul National University Hospital Biomedical Research Institute, Seoul, Korea (the Republic of); 5 Disaster Medicine Research Centre, Seoul National University Medical Research Centre, Seoul, Korea (the Republic of); 6 Urban Planning and Engineering, Yeungnam University, Gyeongsan-si, Korea (the Republic of); 7 Department of Environmental Planning, Seoul National University, Gwanak-gu, Korea (the Republic of); 8 Emergency Medicine, Seoul National University Hospital, Seoul, Korea (the Republic of); 9 Institute of Health Policy and Management, Medical Research Center, Seoul National University, Seoul, Korea (the Republic of)

**Keywords:** Ecological Study, Environmental Modification, Policy, Public Health, Motor vehicle - Occupant

## Abstract

**Background:**

Frozen road conditions could be a factor in road traffic injuries, and seasonality and urbanisation level are potential influencing factors. However, few studies have considered this relationship. Therefore, we examined the effect of frozen road conditions on road traffic injury rates and the differences across seasons and urbanisation levels.

**Methods:**

We used nationwide data on road traffic injuries and weather from the National Emergency Medical Service Run Sheet and the Korea Meteorological Administration, respectively, from 2018 to 2021. We analysed the relationship between frozen road conditions and road traffic injuries by administrative district and day using a generalised estimating equation with log link function and Poisson distribution, stratified by season and urbanisation level.

**Results:**

After excluding summer, the analysis of 605 254 road traffic injury cases revealed a higher road crash incidence under frozen road conditions, with injuries increasing by 30% (rate ratio (RR): 1.33, 95% CI: 1.30 to 1.37). The relationship between road traffic injury rate and frozen road conditions varied significantly by season and urbanisation level. The stratification analysis showed that the relationship between these two variables was significant in the fall (RR: 2.07, 95% CI: 1.74 to 2.46) and winter (RR: 1.37, 95% CI: 1.34 to 1.40). Furthermore, the road traffic injury rate was higher on frozen road conditions in urban (RR: 1.24, 95% CI: 1.19 to 1.29) and rural areas (RR: 1.47, 95% CI: 1.42 to 1.51), not in metropolitan areas.

**Conclusion:**

The road traffic injury rate increased under frozen road conditions and varied by season and urbanisation level. These findings indicate the need for road safety strategies tailored according to season and urbanisation level.

**WHAT IS ALREADY KNOWN ON THIS TOPIC**

- Road traffic injuries stand as a prominent contributor to the global mortality rate. Weather conditions have been considered as a major factor in road traffic injuries. However, regional differences and seasonal variation have not been considered when examining the effect of weather on road traffic crashes.

**WHAT THIS STUDY ADDS**

- This study examined the impact of frozen road conditions on road traffic crashes across different levels of urbanisation and seasons. It found that frozen road conditions in winter were significantly associated with increased road traffic crashes, with significant differences between metropolitan, urban and rural areas.

**HOW THIS STUDY MIGHT AFFECT RESEARCH, PRACTICE OR POLICY**

- This study highlights increased traffic injuries under frozen road conditions and their variability by season and urbanisation level, indicating the necessity for tailored road safety strategies.

## Background

 Road traffic injuries (RTIs) stand as a prominent contributor to global mortality rate, with the WHO reporting road crashes as a primary cause of death among individuals aged 5–29 years and as the 12th leading cause of mortality across all age groups in 2019.^1^ Among the Organization for Economic Cooperation and Development countries, South Korea (hereafter Korea) had the fourth highest road crash rate.^2^ According to a 2022 report from Statistics Korea, RTI was the second highest external cause of death.^3^

Weather conditions have been considered as a major factor in road traffic crashes and injuries, and precipitation in winter (eg, snow, ice pellets) is a well-known factor for road traffic crashes.^4 5^ However, regional differences and seasonal variation have not been considered when examining the weather effect on RTIs. Urbanisation could influence the weather effect on RTIs because rural areas have different medical service systems and road environments than metropolitan or urban areas. However, prior studies have not compared weather effects on RTIs between urban and rural areas because they were conducted in a region^6 7^ or group of regions,^8 9^ not a country. Similarly, studies on road crash risks in Korea only examined datasets from major cities such as Seoul^10^ and Busan.^11^

Another issue that needs investigation is the effect of season on the association between weather and RTIs. Korea experiences significant climatic variations between summer and winter, with the summer being characterised by heavy rainfall and high temperatures, whereas the winter is extremely cold and dry. Climatic variations in weather effects on RTIs might be evident in Korea. Therefore, to understand the relationship between weather and RTIs in Korea, both seasonal and urban-rural differences must be considered. However, to the best of our knowledge, no research to date has addressed this aspect.

The Korean government operates the Emergency Medical Service (EMS) Run Sheet that includes detailed information on ambulance dispatch and patient condition and management. Consequently, this enables a focused analysis of RTIs that necessitates EMS intervention. A weather database is also provided that encompasses nationwide weather information recorded by automatic weather stations. These datasets allow for a nationwide study to explore regional differences and seasonal variations in road crash patterns in Korea.

This study aims to investigate: (1) the relationship between frozen roads and the RTI rate per 100 000 people, (2) how the association between frozen road conditions and RTI rate varies by season and (3) how the relationship between frozen roads and RTI rate differs based on urbanicity.

## Methods

### Data source

We assessed the relationship between weather and RTI rate using two datasets from the EMS Run Sheet and Automatic Weather Station (AWS). The National Fire Agency of South Korea administers a unified, centralised EMS system. EMS personnel are required to complete a ‘run sheet’ that documents details about the ambulance dispatch and the patient. We defined RTI patients as those whose cause of ambulance dispatch on the EMS Run Sheet was recorded as ‘traffic crashes’. Using the EMS Run Sheet database, we identified 834 637 RTI patients between 2018 and 2021; 801 945 patients were included in the analysis after excluding those with missing information on age (n=5127), sex (n=900) or weather conditions (n=26 665). The Korea Meteorological Administration collects AWS data, including mean temperature, wind speed and rainfall measured at 523 stations distributed nationwide (Korea Meteorological Administration). AWS data were recorded daily from 2018 to 2021, and we used weather information collected between 1 January 2018 and 31 December 2021. Because of the higher number of weather stations in some administrative districts, we computed the means for the AWS data of each administrative district. For example, Gwanak-gu, Seoul, has three stations; therefore, we calculated the weather information of Gwanak-gu by averaging the data from all three. If any administrative districts did not have an AWS, we used the average of the adjacent administrative districts. This study uses secondary datasets.

### Patient and public involvement

Patients and/or the public were not involved in the design, conduct, reporting or dissemination plans of this research.

### Outcome variable

The outcome variable was RTI rate, standardised per 100 000 people by both age and sex. To estimate the standardised rate, we classified age into eight groups (ie, 0–19, 20–29, 30–39, 40–49, 50–59, 60–69, 70–79 and ≥80 years) and sex into two groups (male and female) for 229 administrative districts. We created the strata based on age group and sex and calculated the strata-specific crude RTI rate per 100 000 people using the resident registration population (Korean Statistical Information Service) for each region. We estimated the standardised RTI rate by multiplying the crude rate and proportion of people in each stratum.

### Exposure variables

We considered frozen road conditions as exposure variables. Previous research suggests that the type of winter precipitation (snow, ice pellets, freezing rain) makes no significant difference in crash risks^12^; thus, we focused on frozen road conditions, which are considered to have a more direct effect on crash occurrence. Frozen road conditions were defined as precipitation with mean temperatures under 0°C and divided into two groups (days with and without frozen road conditions).

### Covariates

We included mean wind speed, year, season and urbanisation level as possible confounders in the relationship between weather conditions and RTIs. Mean wind speed (m/s) was similarly assessed. The study period was categorised into four distinct years: 2018, 2019, 2020 and 2021. Seasons were classified into four groups: spring (March–May), summer (June–August), fall (September–November) and winter (December–February). Urbanisation level was determined based on South Korea’s administrative division classification of three categories: metropolitan cities (comprising one special city, six metropolitan cities and one self-governing city), urban areas (designated as ‘Si’ in administrative terms) and rural areas.

### Analysis

To identify the trend of RTIs, we estimated the age-standardised and sex-standardised RTI mean rate per 100 000 people by urbanisation level, season and year, and plotted the daily RTI rate. Owing to the absence of frozen road conditions in summer, we excluded this season from the association analysis and focused on the relationship between frozen road conditions and RTIs in seasons where such conditions are possible (n=605,254). The RTI rate on the current day could be correlated with the RTI rate yesterday in the same districts. To consider the correlation of outcomes between days, we applied the generalised estimating equation with exchangeable correlation, log link function and Poisson distribution.


$$Log\left(E\left(Y\right)\right)=\begin{array}{l} \beta_{0}+\beta_{1}\;frozen\;road\;conditions_{ij}\\ +\beta_{2}\;mean\;wind\;speed_{ij}\\ +\beta_{3}\;2019_{j}+\beta_{4}\;2020_{j}+\beta_{5}\;2021_{j}\\ +\beta_{6}\;fall_{j}+\beta_{7}\;winter_{j}\\ +\beta_{8}\;urban\;areas_{i}+\beta_{9}\;rural\;areas_{i} \end{array}$$

Administrative district and date were defined as *i* and *j*, respectively, and the age-standardised and sex-standardised RTI rate was the expected value of *Y*. We also performed stratification analysis according to urbanisation level and season. We investigated the interaction between frozen road conditions, season and urbanisation level to assess how the association between weather conditions and RTI rate varies by season and urbanisation level. For the sensitivity analysis, we performed stratification to examine seasonal variations in the relationship between frozen road conditions and RTIs across different levels of urbanisation. Furthermore, we examined the association between weather conditions and RTI rate after revising the criterion of precipitation to 10 mm per day. All analyses were performed using STATA/MP V.17.0 (Stata Corp., College Station, Texas, USA). Results from the association analyses are presented as rate ratios (RRs) with 95% CIs.

## Results

**Figure 1:**
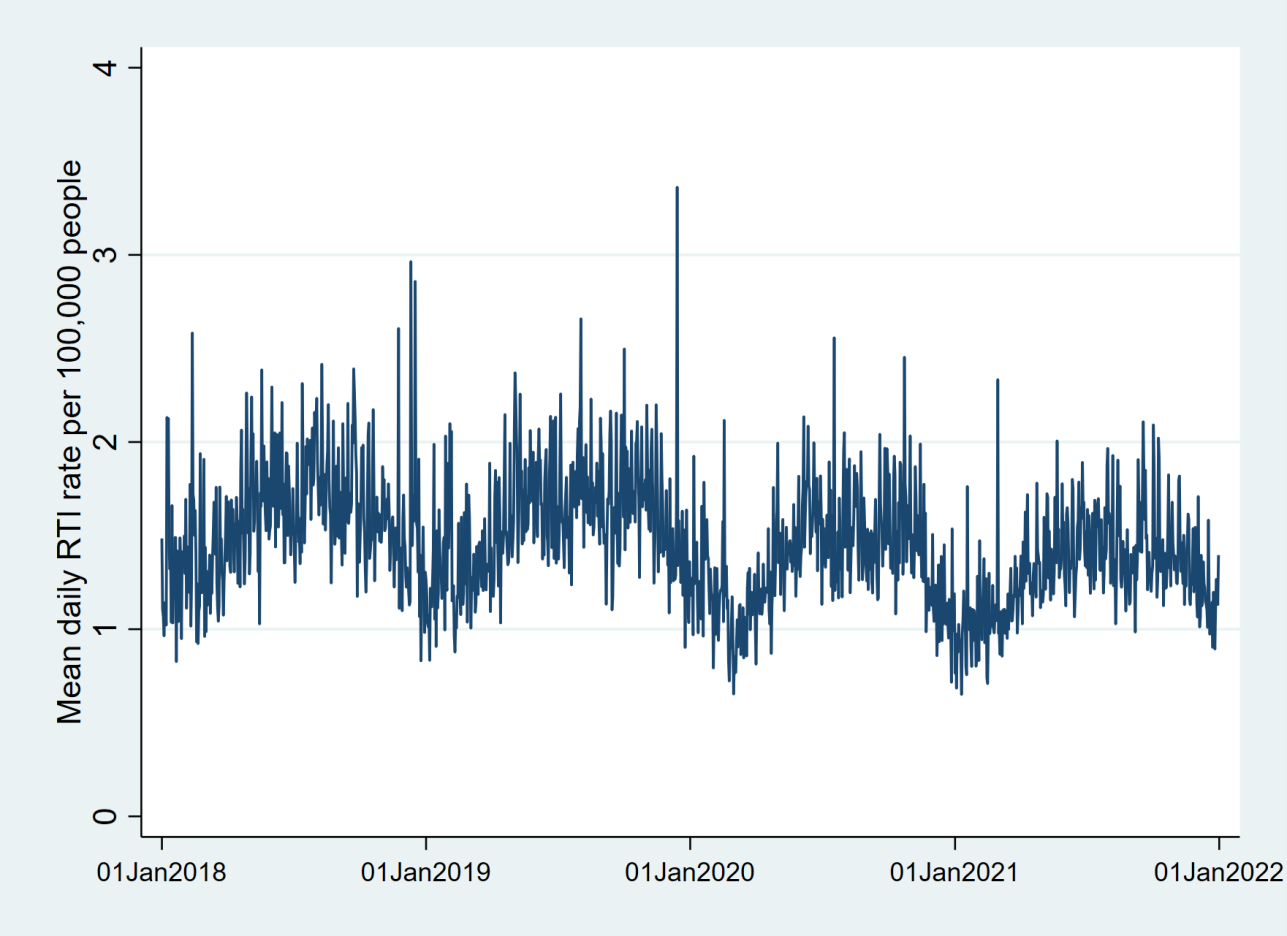
Mean daily road traffic injury rate per 100 000 people between 2018 and 2021 in South Korea.

Table 1 shows the age-standardised and sex-standardised RTI rate per 100 000 people in South Korea from 2018 to 2021. The overall RTI rate was 1.46 per 100 000 people compared with 1.79 per 100 000 people under frozen road conditions. The RTI rate was higher in rural areas (2.04) and during the summer (1.63). Mean daily RTI rate showed a decreasing trend over the observed period (figure 1).

**Table 1:** Number of standardised road traffic injuries per 100 000 people between 2018 and 2021 in South Korea

	Total (n=3 34 569 days)		Frozen road days (n=4391 days)	
	Mean	SD	Mean	SD
Total	1.46	2.43	1.79	3.48
Urbanisation level				
Metropolitan	0.96	1.07	0.91	1.22
Urban	1.38	1.46	1.50	2.30
Rural	2.04	3.70	2.42	4.63
Season				
Spring	1.39	2.25	1.48	2.95
Summer	1.63	2.61		
Fall	1.57	2.44	4.07	5.68
Winter	1.26	2.38	1.78	3.47
Year				
2018	1.58	2.50	2.39	4.15
2019	1.58	2.62	2.10	3.64
2020	1.37	2.31	1.62	3.75
2021	1.31	2.26	1.32	2.53

After excluding summer, the RTI rate on days with frozen road conditions (95% CI: 1.30 to 1.37) was 1.33 times higher compared with days without such conditions, after adjusting for covariates such as urbanisation level and season (table 2). This positive association between frozen road conditions and RTI rate was statistically significant only in the fall (RR: 2.07, 95% CI: 1.74 to 2.46) and winter (RR: 1.37, 95% CI: 1.34 to 1.40; table 3). Significant differences were found in the relationships between spring and both fall (p value for interaction term: <0.001) and winter (p value for interaction term: <0.001). Furthermore, a higher RTI rate was observed on days with frozen road conditions compared with days without in both urban (RR: 1.24, 95% CI: 1.19 to 1.29) and rural areas (RR: 1.47, 95% CI: 1.42 to 1.51). There were significant differences in the associations between urban and metropolitan areas (p value for interaction term 0.004) as well as between urban and rural areas (p value for interaction term <0.001).

**Table 2:** Association between weather and road traffic injuries between 2018 and 2021 in South Korea

	Road traffic injury rate per 100 000 people		
	Mean (SD)	Rate ratio	95% CI
Day without frozen road conditions	1.40 (2.3)	ref	
Day with frozen road conditions	1.79 (3.5)	1.33*	(1.30 to 1.37)

Adjusted for mean wind speed, year, season and urbanisation level.

*P value <0.001.

**Table 3:** Association between weather and road traffic injuries by season and urbanisation level between 2018 and 2021 in South Korea

	Road traffic injury rate per 100 000 people		
	Rate ratio	95% CI	P value for interaction term
Season†			
Spring	0.90	(0.79 to 1.02)	ref
Fall	2.07*	(1.74 to 2.46)	<0.001
Winter	1.37*	(1.34 to 1.40)	<0.001
Urbanisation level‡			
Metropolitan	1.05	(0.98 to 1.12)	0.004
Urban area	1.24*	(1.19 to 1.29)	ref
Rural area	1.47*	(1.42 to 1.51)	<0.001

*P value <0.001.

†Adjusted for mean wind speed, year and urbanisation level.

‡Adjusted for mean wind speed, year and season.

The results also revealed an association between frozen road conditions and RTI rates across seasons and urbanisation levels (online supplemental material 1). Frozen road conditions in winter were significantly associated with increased RTI rate in metropolitan (RR: 1.08, 95% CI: 1.01 to 1.16), urban (RR: 1.26, 95% CI: 1.20 to 1.31) and rural (RR: 1.52, 95% CI: 1.47 to 1.57) areas. Significant differences were found in the associations between urban and both metropolitan cities (p value for interaction term: 0.002) and rural areas (p value for interaction term <0.001) in winter. In the sensitivity analysis, the association between frozen road conditions and RTI rates was statistically significant only in rural areas (RR: 2.01, 95% CI: 1.65 to 2.44) during winter, but not in metropolitan and urban areas (online supplemental material 2).

## Discussion

We examined how frozen road conditions affect RTI rates nationwide in Korea using EMS run sheet and administrative weather data. The results show an association between frozen road conditions, characterised by a combination of freezing temperatures and precipitation, and increased RTI risk. Overall, the RTI rate increased by approximately 30% on days with frozen road conditions compared with days without. Furthermore, the impact of weather conditions on RTI risk varied significantly depending on the season and urbanisation levels. In fall, RTIs were more than two times as likely under frozen road conditions.

Our findings indicate a significant increase in RTI rates under frozen road conditions in Korea. Several studies have previously reported an association between traffic injuries and colder temperatures^6 10 13^ or winter precipitation.^12 14^ Lee *et al*^10^ investigated the relationship between road crashes and temperature in Seoul, finding that when the temperature fell below −5.7°C, crashes increased by 2.1% for every 1°C decrease. Another study analysing the national emergency database in Korea reported an increased relative risk of unintentional crashes when temperatures dropped below 0°C.^15^ Juga *et al*^16^ reported an increasing trend in road crashes in Helsinki related to daily precipitation and low temperatures; however, they did not consider the combined effect of precipitation and temperatures. The impact of winter precipitation on RTIs has been well documented in several studies.^6 12 14 17^ Winter precipitation, including snowfall and ice pellets, occurs at low temperatures, could lead to frozen road conditions and could increase crash rates by reducing road surface friction. RTIs may also increase on frozen roads under a similar mechanism. To prevent RTIs, preparations for de-icing when temperatures are below 0°C and measures to reduce traffic in high-risk areas are necessary.

Notably, the highest increase in the RTI rate occurred under frozen road conditions in fall. This increase can likely be attributed to driver inattention. Johansson *et al*^18^ reported higher road crash rates in southern Sweden compared with northern Sweden, which they explained as drivers tending to be less prepared for frozen road conditions in southern Sweden due to the lower frequency of such conditions and lower temperatures in that region. Black and Mote^12^ and Norman *et al*^19^ found that road crash rates increased from the onset of snowfall until drivers adapted to the new road conditions. Therefore, a reasonable expectation could be that, since drivers do not anticipate frozen road conditions in fall, they are less likely to take compensatory measures such as reduced speed and increased alertness. This lack of preparation may contribute to the much higher RTI rates observed. A similar trend was observed in Japan, where road crashes were reported to peak in early winter.^20^ Furthermore, local government authorities responsible for road maintenance may potentially pay less attention to preventing and mitigating frozen road conditions during the fall compared with measures taken in the winter. To reduce RTIs in fall, measures are needed to prevent crashes when below-freezing temperatures are combined with precipitation.

A significant finding of our study is variation in RTI rates according to urbanisation level. Under frozen road conditions, rural areas exhibited higher RTI rates compared with metropolitan cities, and this trend was consistent during winter. Several studies have addressed the regional differences in the effects of weather on crash rates. Edwards^21^ reported higher crash rates in rural areas across all weather conditions compared with urban cities in England and Wales, suggesting that urban areas have lower crash rates due to superior infrastructure, including better street lighting and road maintenance. Rural areas may experience higher injury rates due to higher average speeds and poorer road conditions. A meta-analysis^22^ found that the fatal road crash rate was 3.2 times higher in rural areas in Canada and Australia. Huang *et al*^23^ analysed emergency medical system records in Taiwan and noted that trauma-related calls were more frequent in rural compared with urban areas. These findings are consistent with our results, showing that RTI rates were consistently higher in rural areas across fall and winter when compared with metropolitan cities. A study of risk factors for the severity of road crashes on frozen roads in Korea discovered that severe road crashes were more common in outskirts than in city centres.^24^ Rural areas may be at a higher risk for RTI incidents related to frozen road conditions because of their geographical conditions and poorer ability to prevent frozen roads. Thus, preventive measures may be necessary, such as increased emergency vehicle presence or enhanced road maintenance during winter.

This study has several limitations that should be considered. First, because we conducted our analysis using daily weather records, establishing a direct causal relationship between daily weather conditions and RTI rate is challenging. For example, frozen road conditions could occur after an RTI. In addition, using weather information for administrative districts may have created measurement errors for weather conditions. Second, it is important to note that the EMS Run Sheet data do not encompass entire RTIs in Korea, as some individuals may have sought medical help without the assistance of EMS. Additionally, the injury severity was not considered when defining the RTI from the EMS run sheet database. However, moderately to severely injured patients who require emergency care are typically transported to the emergency department by EMS in Korea.^25^ Therefore, the RTI rate obtained could potentially represent a significant number of cases nationally. Finally, some confounders were not included, such as traffic volume. These limitations should be considered when interpreting the results of our study.

## Conclusion

Most previous studies on the association between weather conditions and RTIs in Korea were conducted in specific regions or roads.^10 26–28^ However, this study examined the association between frozen road conditions and RTI rates requiring ambulance dispatch using nationwide datasets from the EMS Run Sheet and AWS. Furthermore, this study is the first to investigate the effects of season and urbanisation level on this relationship. Our results showed that the incidence of traffic injuries increased on frozen road conditions and could vary by season and urbanisation level. These findings indicate the need for road safety strategies to be tailored according to the season and urbanisation level.

## Supplementary material

10.1136/ip-2024-045327 online supplemental material 1

10.1136/ip-2024-045327 online supplemental material 2

## Data Availability

Data are available upon reasonable request.
