# Supplementary material for: Effects of urbanisation and seasons on the relationship between frozen road conditions and road traffic injury: a longitudinal study of national emergency medical service data in South Korea
Source: Inj Prev. 2024 Dec 4;32(4):e045327. doi: 10.1136/ip-2024-045327 (PMC13422108; doi:10.1136/ip-2024-045327)
Supplement: online supplemental material 2 [file ip-32-4-s002.pdf]

Supplementary material 2. The association between weather and road traffic injuries by urbanisation across seasons in South Korea after revising the criterion of precipitation to 10mm per day

Season	Region	Road traffic injury rate per 100,000 people		
		Frozen road days		
		Rate ratio	95% CI	p-value for interaction term
Spring	Metropolitan	.	.	.
	Urban area	1.85	(0.46, 1.56)	ref
	Rural area	2.24***	(1.67, 3.01)	0.006
Fall	Metropolitan	.	.	.
	Urban area	.	.	.
	Rural area	.	.	.
Winter	Metropolitan	1.13	(0.70, 1.84)	0.514
	Urban area	0.91	(0.62, 1.35)	ref
	Rural area	2.01***	(1.65, 2.44)	0.001

Adjusted for mean temperature, mean wind speed, and year.

* p-value<0.05

** p-value<0.01

*** p-value<0.001
